# Vertebrate Lrig3-ErbB Interactions Occur *In Vitro* but Are Unlikely to Play a Role in Lrig3-Dependent Inner Ear Morphogenesis

**DOI:** 10.1371/journal.pone.0008981

**Published:** 2010-02-01

**Authors:** Victoria E. Abraira, Takunori Satoh, Donna M. Fekete, Lisa V. Goodrich

**Affiliations:** 1 Department of Neurobiology and Program in Neuroscience, Harvard Medical School, Boston, Massachusetts, United States of America; 2 Division of Biological Science, Graduate School of Science, Nagoya University, Nagoya, Aichi, Japan; 3 Department of Biological Sciences, Purdue University, West Lafayette, Indiana, United States of America; Medical College of Georgia, United States of America

## Abstract

**Background:**

The *Lrig* genes encode a family of transmembrane proteins that have been implicated in tumorigenesis, psoriasis, neural crest development, and complex tissue morphogenesis. Whether these diverse phenotypes reflect a single underlying cellular mechanism is not known. However, Lrig proteins contain evolutionarily conserved ectodomains harboring both leucine-rich repeats and immunoglobulin domains, suggesting an ability to bind to common partners. Previous studies revealed that Lrig1 binds to and inhibits members of the ErbB family of receptor tyrosine kinases by inducing receptor internalization and degradation. In addition, other receptor tyrosine kinase binding partners have been identified for both Lrig1 and Lrig3, leaving open the question of whether defective ErbB signaling is responsible for the observed mouse phenotypes.

**Methodology/Principal Findings:**

Here, we report that Lrig3, like Lrig1, is able to interact with ErbB receptors *in vitro*. We examined the *in vivo* significance of these interactions in the inner ear, where *Lrig3* controls semicircular canal formation by determining the timing and extent of *Netrin1* expression in the otic vesicle epithelium. We find that ErbB2 and ErbB3 are present in the early otic epithelium, and that Lrig3 acts cell-autonomously here, as would be predicted if Lrig3 regulates ErbB2/B3 activity. However, inhibition of ErbB activation in the chick otic vesicle has no detectable effect on *Netrin* gene expression or canal morphogenesis.

**Conclusions/Significance:**

Our results suggest that although both Lrig1 and Lrig3 can interact with ErbB receptors *in vitro*, modulation of Neuregulin signaling is unlikely to contribute to Lrig3-dependent processes of inner ear morphogenesis. These results highlight the similar binding properties of Lrig1 and Lrig3 and underscore the need to determine how these two family members bind to and regulate different receptors to affect diverse aspects of cell behavior *in vivo*.

## Introduction

**Figure 1:**
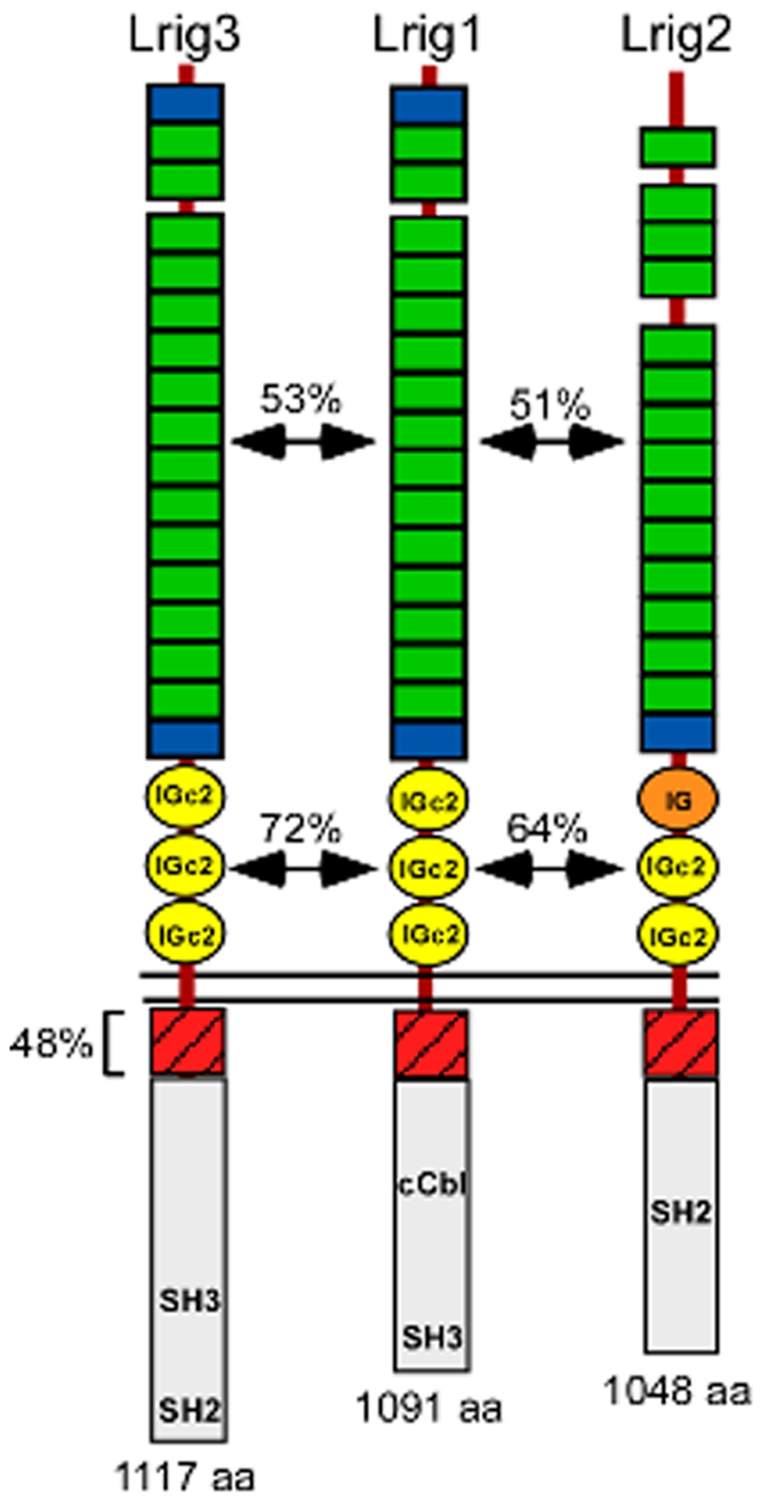
Lrig family protein structures. Alignment of Lrig1, Lrig2, and Lrig3 shows that Lrig1 and Lrig3 share the same number and spacing of Leucine Rich Repeats (LRRs, green) and Immunoglobulin domains (Ig, yellow). In contrast, Lrig2 lacks an N' terminal LRR (blue) and has a different kind of Ig repeat, as reflected in the lower degree of identity with Lrig1. In the intracellular domains, all three family members share a highly conserved (48% identical) stretch of 48 amino acids (bracketed). Apart from this motif and some putative SH2 and/or SH3 domains, the cytoplasmic tails vary widely both in length and composition. Lrig1 is the only family member with a confirmed ability to bind c-Cbl. Arrows indicate degrees of identity between the LRR domain-containing regions and the Ig domain-containing regions. The amino acid (aa) length of each protein is also indicated. Domain structure was obtained with SMART protein (http://smart.embl-heidelberg.de/) and putative intracellular domains were defined by Scansite (http://scansite.mit.edu/).

The mammalian genome contains an expanded repertoire of transmembrane proteins that carry both leucine-rich repeats (LRR) and immunoglobulin (Ig) domains in their extracellular domains [1]. Within this LRR-Ig superfamily, only the Lrig subfamily contains both invertebrate and vertebrate orthologs, represented by Dlig1 (also called Lambik) in flies and Lrig1, Lrig2, and Lrig3 in vertebrates. Lrig proteins have large extracellular domains with either sixteen (Lrig1 and Lrig3) or fifteen (Lrig2) LRRs and three Ig domains (Fig. 1). Based on a distant similarity to Kekkons, which are the only large family of LRR-Ig proteins in flies [2], [3], Lrig proteins have been studied mostly as putative regulators of ErbB signaling pathways [4]–[6]. There are four ErbB receptors: ErbB1, which binds EGF and is thus better known as the EGF receptor (EGFR), and ErbB2-4, which bind Neuregulin (NRG) ligands [7]. Upon activation, ErbB receptors form homophilic and heterophilic dimers, become phosphorylated, and go on to induce a myriad of cellular processes, including cell proliferation, differentiation, and survival in glia, neurons, and mesoderm and in systems as diverse as the heart, lung, and mammary gland [8]–[13].

There is considerable evidence that Lrig1 is a negative regulator of ErbB signaling. Human Lrig1 is induced by EGF signaling and antagonizes downstream signaling events by binding the extracellular domain of all four ErbB receptors and by enhancing receptor ubiquitination and subsequent degradation [4]–[6]. Lrig1 interacts with ErbB receptors via its extracellular domain, while degradation seems to involve the recruitment of the E3 ubiquitin ligase c-Cbl to a site in the cytoplasmic domain of Lrig1 [5]. Consistent with its role in ErbB receptor degradation *in vitro*, *Lrig1* is deleted in several different forms of human cancers, and low expression of *Lrig1* correlates with poor prognosis of cervical and breast cancer [14]–[20]. Whether or not other Lrig family members also bind to ErbB receptors has not been examined. However, both Lrig2 and Lrig3 have also been implicated in human cancer [21]–[23].

Although studies of *Lrig* mutant phenotypes confirm the importance of Lrig signaling for normal development, the observed defects do not point to an obvious common underlying cellular function. *Lrig1* mutant mice suffer from a psoriasis-like skin disorder of unknown origin [24]. This phenotype correlates with the presence of *Lrig1* in the basal cells of the epidermis and hair follicles and with the prominent role of EGF in keratinocyte differentiation and proliferation [25], [26]; however, specific changes in ErbB receptor activation or distribution have not been reported. *Lrig3* mutant mice, on the other hand, exhibit obvious developmental defects that manifest as abnormal morphogenesis of the inner ear and craniofacial structures [27]. The inner ear defect is caused by expanded expression of the secreted protein Netrin1, a known regulator of inner ear development [28]. How the Lrig3 transmembrane molecule ultimately induces changes in gene expression is not known. Hence, it remains unclear whether the varying collection of defects observed in *Lrig1* and *Lrig3* mutant mice are due to changes in ErbB receptor signaling.

There are several reasons to believe that like Lrig1, Lrig3 could also regulate ErbB signaling. First, Lrig1 is known to interact with ErbB family members through its extracellular domain, which is highly conserved in Lrig3. In addition, the intracellular tail of Lrig3 contains putative SH2 and SH3 domains that have the potential to bind to activated ErbB receptors. Second, *Lrig1* and *Lrig3* expression overlap extensively during development, suggesting a possible functional overlap [27]. Third, despite this broad expression, both *Lrig1* and *Lrig3* mutant mice display relatively mild phenotypes. Strikingly, the *Lrig3* mutant phenotypes occur in regions where *Lrig3* but not *Lrig1* is expressed, namely the lateral canal epithelium of the inner ear and the branchial arches. Thus, Lrig1 may compensate for Lrig3 to regulate ErbB signaling in other regions of the animal. Indeed, like *Lrig1* and *Lrig3*, *ErbB2-4* are also expressed during cochlear maturation [29], [30]. Finally, ErbB signaling plays a crucial and well-established role in complex morphogenesis of other organ systems, suggesting a possible role for the ErbB receptors in canal formation [8], [10], [31].

Based on the close homology between Lrig1 and Lrig3, their overlapping expression patterns, and the known importance of ErbB signaling during development, we hypothesized that Lrig3 modulates the Neuregulin pathway in the inner ear. To test this idea, we performed experiments that address three basic questions: 1) Can Lrig3 interact with ErbB receptors *in vitro*? 2) Are ErbB receptors expressed during inner ear development? and 3) Is ErbB signaling necessary for canal morphogenesis? Our results indicate that although Lrig3 can interact with ErbB receptors *in vitro*, reduction of Neuregulin signaling *in vivo* does not cause any detectable changes in *Netrin* gene expression or in the structure of the inner ear. These results add to a growing body of evidence that Lrig proteins exert their effects through multiple signaling pathways with diverse roles in development and disease.

## Results

### Lrig3 Can Bind ErbB Receptors *In Vitro*

Lrig1 and Lrig3 are closely related proteins, sharing the same number and arrangement of LRRs and Ig domains in the ectodomain (Fig. 1). The composition of these domains is also strongly conserved, with 53% identity between the LRRs and even higher homology in the Ig domains, which are 72% identical. Lrig2, on the other hand, has one fewer LRR and is rarely expressed in the same tissues as either Lrig1 or Lrig3 [27]. Moreover, Lrig2 shares much less homology in the Ig domains (Fig. 1). We therefore focused on comparisons of Lrig1 and Lrig3, and asked whether Lrig3 behaves like Lrig1 with respect to ErbB binding and protein distribution.

**Figure 2:**
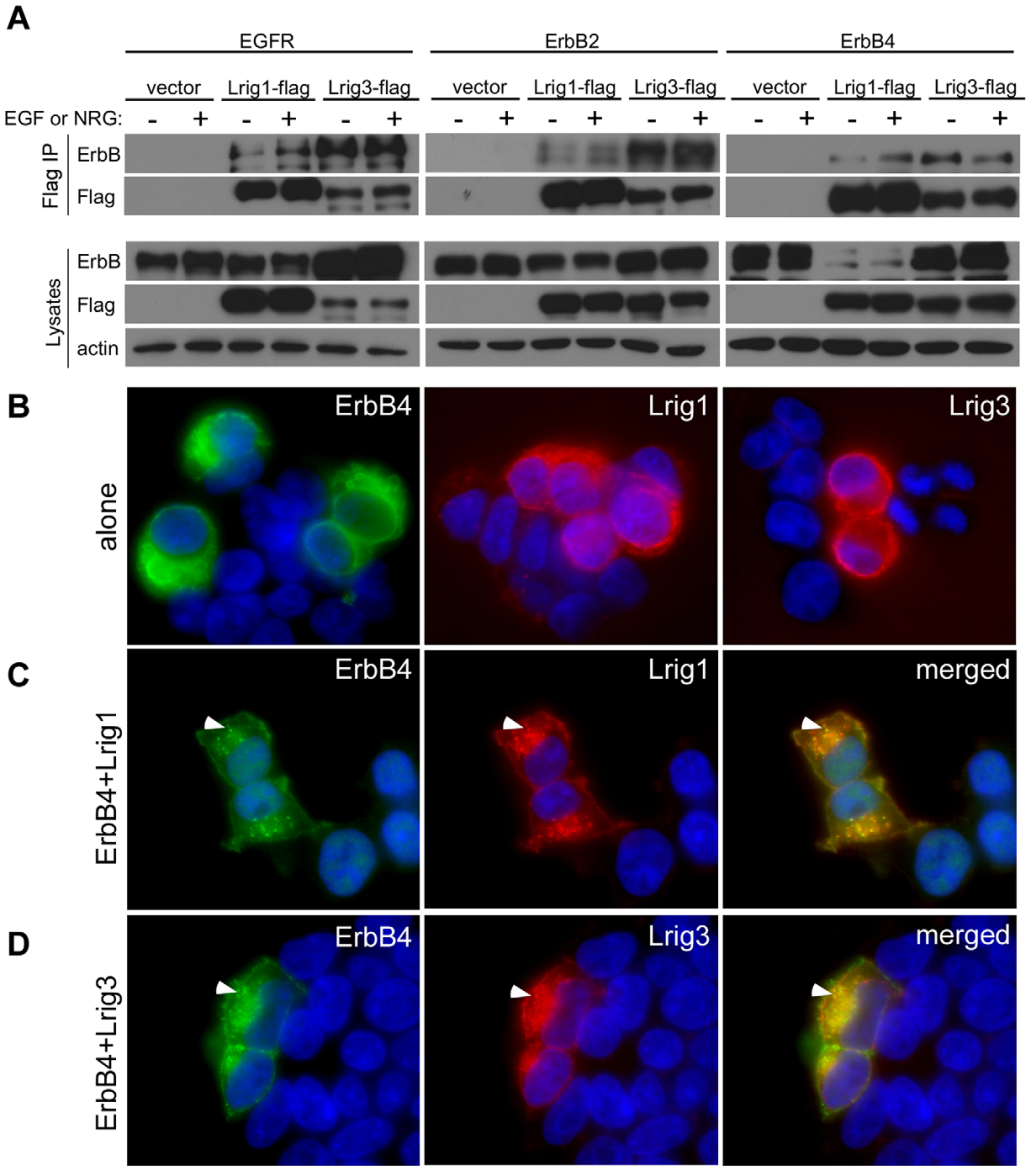
Lrig1 and Lrig3 bind to and co-localize with ErbB receptor tyrosine kinases *in vitro*. (**A**) Like Lrig1, Lrig3 can co-immunoprecipitate with multiple ErbB receptors in HEK293T cells. Lysates were immunoprecipitated with anti-flag antibodies and blotted with anti-ErbB (top) and anti-flag (bottom) antibodies. Both Lrig1-flag and Lrig3-flag can bind to EGFR (left), ErbB2 (middle), and ErbB4 (right) in the absence (−) or presence (+) of ligand (EGF or NRG). No ErbB receptors were precipitated in the absence of epitope-tagged Lrig (vector). Western blotting confirms the presence of each ErbB receptor (ErbB), and epitope-tagged Lrig family member (flag) in total lysates (bottom). Actin was used as a loading control. (**B–D**) Flag and ErbB4 immunocytochemistry on HEK293T cells transfected with ErbB4, Lrig1-flag, and/or Lrig3-flag alone (B) or in combinations (C,D). When expressed on their own, Lrig1, Lrig3 and ErbB4 are detected at the cell surface (B). When either Lrig1 (C) or Lrig3 (D) is expressed in combination with ErbB4, the proteins co-localize in intracellular compartments (arrowheads in C and D).

First, we tested whether the homology between Lrig1 and Lrig3 is sufficient to mediate interactions with ErbB receptors. A series of co-immunoprecipitations were conducted by expressing EGFR, ErbB2, or ErbB4 together with epitope-tagged constructs of Lrig1 or Lrig3 in HEK293T cells, and in the absence or presence of the appropriate ligand. Indeed, like Lrig1, Lrig3-flag co-immunoprecipitates with EGFR, ErbB2 and ErbB4 in lysates from transfected cells (Fig. 2A). ErbB3 was not tested as this family member is kinase-dead and functions as an obligate heterodimer with ErbB2 [7].

Since Lrig3 can to bind to ErbB receptors *in vitro*, we next asked whether, like Lrig1, Lrig3 has the same subcellular distribution as ErbB receptors. We focused on ErbB4 because it is the only NRG receptor that functions as a homodimer. Lrig1-flag, Lrig3-flag, and ErbB4 constructs were expressed either alone or in combination in HEK293T cells; protein localization was determined by immunostaining for the flag epitope and/or for ErbB4. Like both ErbB4 and Lrig1 (Fig. 2A), Lrig3 is present on the cell surface and in intracellular compartments (Fig. 2B). Moreover, both Lrig1 and Lrig3 co-localize with ErbB4 (Fig. 2C,D) Consistent with the role of Lrig1 in ErbB receptor internalization [5], Lrig1 and ErbB4 are present in intracellular vesicles when they are co-expressed (Fig. 2C, arrowhead), with much less protein on the cell surface than when they are expressed independently (Fig. 2A). Similarly, Lrig3 and ErbB4 appear to be enriched in the intracellular pool compared to the cell surface under these conditions (Fig. 2D); however, this effect was not as dramatic as for Lrig1. Although more experiments are needed to elucidate the consequences of Lrig3-ErbB4 co-expression, these results suggest that Lrig1 and Lrig3 share an ability to interact with ErbB receptors *in vitro*.

### Does Lrig3 Act Through ErbB Pathways *In Vivo?*

Although Lrig3 can bind to ErbB receptors through a highly conserved ectodomain, the cytoplasmic domain of Lrig3 bears little homology to Lrig1, leaving open the question of how Lrig3 activity might ultimately influence NRG signaling. To gain insight into this question, we took an *in vivo* approach to determine whether the phenotypes observed in *Lrig3* mutant mice could be due to misregulated ErbB signaling. The most salient defect in *Lrig3* mutant mice occurs in the vestibular apparatus of the inner ear, which normally consists of three semicircular canals oriented with the three dimensions of space. In the absence of *Lrig3*, the lateral semicircular canal is truncated due to increased expression of *Netrin1*, which encodes a secreted extracellular matrix molecule required for canal development [27], [28]. A role for ErbB signaling during canal development has not yet been examined. Therefore, we sought to determine whether Lrig3 acts through ErbB pathways to orchestrate canal morphogenesis.

### ErbB Receptors Are Expressed in the Otic Vesicle Epithelium

**Figure 3:**
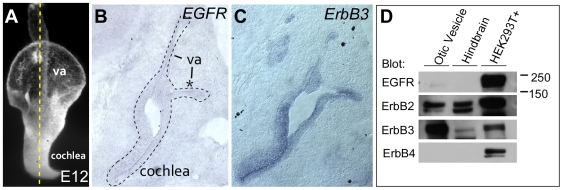
ErbB2 and ErbB3 are present in the developing mouse otic vesicle. (**A**) Paintfill of an E12 mouse otic vesicle depicting plane of section shown in B and C (dashed line). The vestibular apparatus (va) develops dorsally and the cochlea extends ventrally. The lateral canal will develop from the lateral pouch, which is indicated by an asterisk. (**B,C**) *In situ* hybridization of *EGFR* (B) and *ErbB3* (C) on adjacent transverse sections through an E12 mouse head. The otic epithelium is outlined and annotated as in A. Dorsal is up; lateral is right. *EGFR* message is not detectable in the otic vesicle, but is present in other tissues (not shown), confirming that the probe worked. *ErbB3* is expressed throughout the otic vesicle epithelium. (**D**) Western blot of dissected otic vesicle tissue. ErbB2 and ErbB3 are present in abundance, with low levels of EGFR and no detectable ErbB4. HEK293T cells transfected with each ErbB receptor and hindbrain tissue served as controls. Only ErbB2 and ErbB3 are expressed in hindbrain at this stage.

In order to uncover whether Lrig3 acts through ErbB receptors, we first asked whether ErbB receptors are present during inner ear development. The vestibular canals are sculpted from the otic vesicle, a hollow ball of epithelial cells that is formed by invagination of ectoderm at the level of the hindbrain. The lateral canal develops from an epithelial outpocketing of the lateral wall of the otic vesicle at E12 (Fig. 3A). *In situ* hybridization for ErbB receptors in E12 otic vesicles revealed that *EGFR* cannot be detected in the inner ear but that *ErbB3* is broadly expressed throughout the epithelium (Fig. 3B,C). *In situ* probes for other receptors proved to be unreliable and inconclusive. Therefore, to test for the presence of other ErbB receptors, we microdissected otic vesicle epithelia and performed a Western blot analysis with antibodies to all four ErbB receptors (Fig. 3D). As seen by *in situ* hybridization, ErbB3 protein is present in abundance, with only trace amounts of EGFR. ErbB2 protein is also present at high levels, consistent with the fact that ErbB2 and ErbB3 nearly always function as a heterodimer. ErbB4 is not detected. Therefore, we conclude that ErbB2, which cannot bind ligand, and ErbB3, which is kinase-dead, act together to receive NRG signals in the otic epithelium.

### *Lrig3* Acts within the Otic Vesicle Epithelium to Control Fusion Plate Formation

In our model, Lrig3 acts cell-autonomously to control ErbB receptor signaling and hence expression of *Netrin1* in the otic epithelium [27]. In support of this idea, *in situ* hybridization of ErbB3 reveals strong expression in the epithelium but not the surrounding mesenchyme. To determine whether *Lrig3* is required in these ErbB-expressing cells, we generated and analyzed epithelial-specific knock-outs of *Lrig3*.

**Figure 4:**
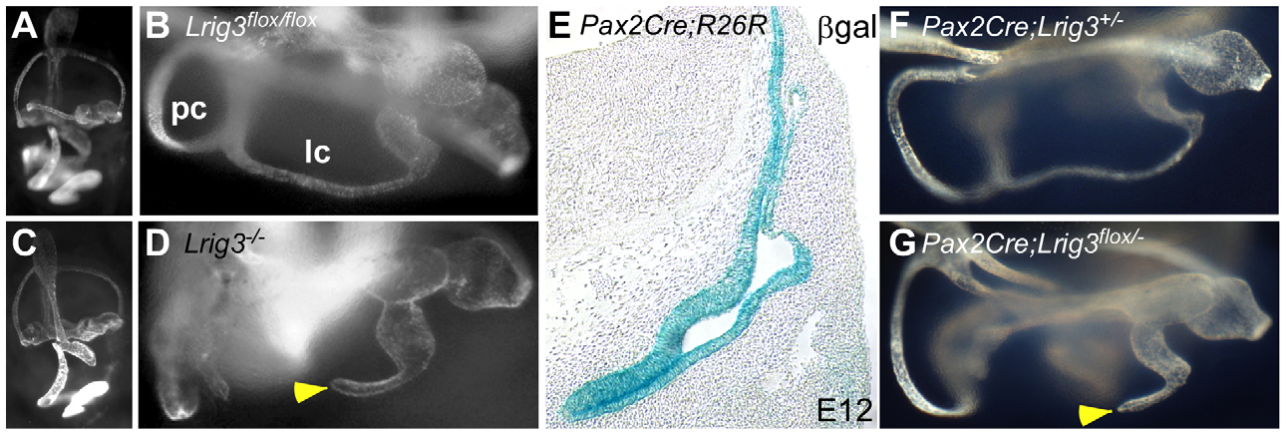
Lrig3 acts within the otic epithelium to regulate canal morphogenesis. (**A,B**) Lateral (A) and top-down (B) views of a paintfilled E14 *Lrig3^flox/flox^* inner ear reveal normal formation of all structures, including the posterior (pc) and lateral canals (lc). (**C,D**) Lateral (C) and top-down (D) views of a paintfilled E14 *Lrig3* null inner ear show a truncation of the lateral canal (arrowhead). (**E**) X-gal staining of a transverse section through an E12 *Pax2Cre;R26R* embryo. βgalactosidase activity is restricted to the otic vesicle epithelium. (**F,G**) Paintfills of E14 inner ears derived from a *Pax2Cre* cross to the *Lrig3^flox^* allele. *Pax2Cre;Lrig3^flox/-^* embryos develop a lateral canal truncation (arrowhead) identical to *Lrig3* nulls (D). Thus, loss of *Lrig3* from Pax2-positive cells in the otic epithelium recapitulates the null phenotype.

Previously, a conditional *Lrig3* allele was generated by flanking the ATG-bearing exon of *Lrig3* with LoxP sites [27]. Null mutant mice exhibit lateral canal truncations and craniofacial defects, consistent with the complete loss of *Lrig3* messenger RNA (Fig. 4C,D and data not shown). To specifically delete *Lrig3* from otic epithelium, *Lrig3^flox^* conditional mice were crossed to mice carrying a *Pax2Cre* transgene, which induces recombination of a β-galactosidase reporter gene throughout the epithelium (Fig. 4E) [32]. Deletion of *Lrig3* from canal epithelial cells results in the same lateral canal truncation evident in hypomorphic and null alleles of *Lrig3* (Fig. 4G). Thus, Lrig3 acts within the otic epithelium to coordinate canal morphogenesis.

### Expression of Dominant-Negative ErbB in the Chick Otic Vesicle Has No Effect on Canal Morphogenesis

Our results indicate that Lrig3 can bind to ErbB receptors and that ErbB2/B3 are present in the otic epithelium, where Lrig3 acts cell autonomously to control canal formation. However, while Lrig1 is known to inhibit ErbB signaling, whether or not Lrig3 has the same molecular effects is unclear. If Lrig3 normally inhibits ErbB activity, then decreased ErbB signaling is predicted to lead to a loss of *Netrin1* expression and hence arrested canal formation, as occurs in *Netrin1* mutant mice. Alternatively, if Lrig3 potentiates ErbB activity, we would predict canal truncations similar to what is seen in *Lrig3* mutant mice. To distinguish between these possibilities, we performed an unbiased experiment to ask whether ErbB signaling is required for any aspect of canal morphogenesis.

Several different *ErbB* mouse mutants are available and have been studied extensively [33]-[38]. However, most mutant mice die at early embryonic stages due to various defects in cardiac development and are therefore not useful for the study of inner ear morphogenesis [39]–[42]. Therefore, we turned instead to the chick as a model system for canal morphogenesis. The mouse and chicken inner ear develop similarly both at the cellular and molecular level [43], [44]. However, the chicken is more accessible and amenable to manipulations than the mouse. Indeed, the chick has been used successfully in the past for the study of both FGFs and BMPs in inner ear morphogenesis [45], [46].

**Figure 5:**
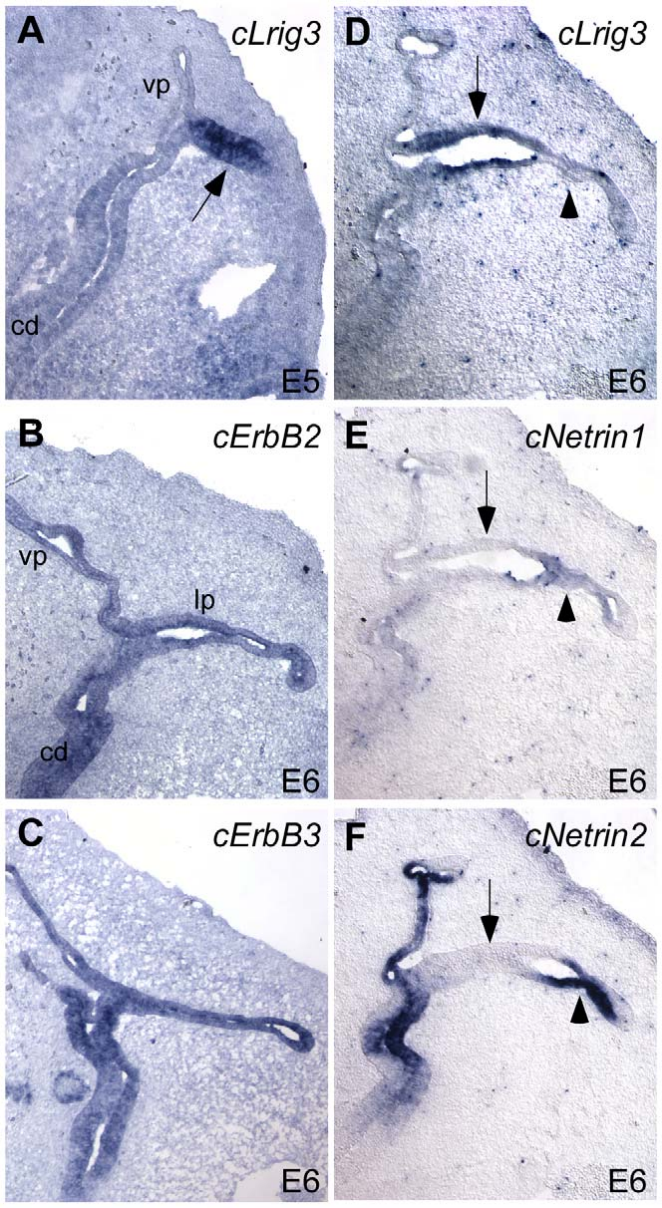
Expression of *cLrig3*, *cNetrin1*, *cNetrin2*, *cErbB2* and *cErbB3* in chick otic epithelium. (**A–F**) *In situ* hybridization of *cLrig3* (A,D), *cErbB2* (B), *cErbB3* (C), *cNetrin1* (E), and *cNetrin2* (F) on transverse sections of E5 (A) or E6 (B–F) chicken heads. Dorsal is up; lateral is right. As in the mouse, chicken *Lrig3* is initially present throughout the lateral pouch at E5 (arrows, A), and then sustained in the non-fusing epithelium (arrow, D) but not the fusion plate (arrowhead, D) at E6. Hybridization of adjacent sections confirms that chicken *Netrin1* expression is complementary to *Lrig3*, with transcript detected in the fusing (arrowhead, E) but not non-fusing epithelium (arrow, E). In addition, *cNetrin2*, which does not exist in mice, is robustly expressed overlapping with *cNetrin1* in the fusion plate (arrowhead, F). Both *ErbB2* and *ErbB3* are transcribed throughout the otic epithelium (B) including the vertical pouch (vp), lateral pouch (lp) and cochlear duct (cd).

First, we confirmed the presence of *Lrig3* in the chicken inner ear by *in situ* hybridization of embryonic day 5 (E5) embryos, which is comparable to E12 in mouse. As predicted, *Lrig3* is transcribed in the lateral pouch epithelium of the developing chick as it is in mice, suggesting a common function in both species (Fig. 5A,D). Similarly, chicken ErbB2 and ErbB3 are expressed broadly throughout the otic epithelium as in mice (Fig. 5B,C). Finally, we confirmed expression of two closely-related *Netrin* genes, *cNetrin1* and *cNetrin2*, which overlap extensively in the chick embryo [47], [48]. Notably, there is no mouse ortholog of *Netrin2*, but in chicks, Netrin activity appears to reflect contributions from both Netrin1 and Netrin2. Consistent with this idea, we found that both *cNetrin1* and *cNetrin2* are present in the fusion plate epithelium, with *cNetrin1* expressed slightly later and at lower levels (Fig. 5E) than *cNetrin2*, which appears to be the major player in chicken (Fig. 5F). As in mouse, this expression is complementary to *cLrig3*, which is downregulated in the presumptive fusion plate and maintained only in the non-fusing epithelium at E6 (Fig. 5D–F).

**Figure 6:**
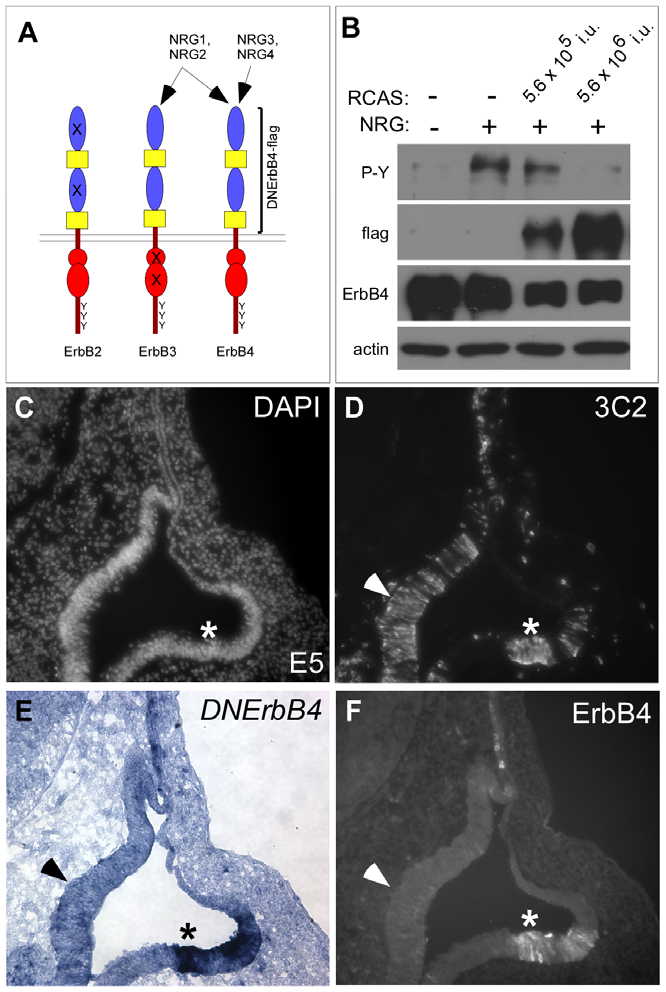
RCAS(A)/DNErbB4-flag produces DNErbB4-flag protein *in vitro* and *in vivo*. (**A**) Diagrams of ErbB2-4 receptors depicting their extracellular domains (blue and yellow), intracellular kinase modules (red), and tyrosine residues (Y). Four Neuregulin ligands (NRG1-4) bind to ErbB3 and ErbB4 as shown. Since ErbB2 cannot bind ligand and ErbB3 is kinase dead, ErbB2 and ErbB3 typically heterodimerize in order to bind and transduce Neuregulin signals. Dominant negative (DN) ErbB4 consists of the extracellular and transmembrane domains of the human ErbB4 receptor (bracketed), which binds to all four NRG ligands and prevents them from interacting with their endogenous receptors. Therefore, DNErbB4 effectively blocks Neuregulin signaling through all ErbB receptors. (**B**) Western blot analysis of lysates of DF1 chicken cells transiently transfected with ErbB4 and infected with RCAS(A)/DNErbB4-flag in the presence or absence of NRG, as indicated. Production of ErbB4 was confirmed by probing lysates with antibodies to ErbB4 (third row). Antibodies against the flag epitope reveal the relative amount of DNErbB4-flag in cells infected with 5×10^5^ or 5×10^6^ infectious units of RCAS(A)/DNErbB4-flag (second row). NRG stimulation of uninfected cells induces phosphorylation of ErbB4, as detected by blotting with anti-phosphotyrosine (P-Y) antibodies (top). This phosphorylation is reduced in the presence of increased levels of DNErbB4-flag. Actin served as a loading control for the P-Y blot (bottom). (**C–F**) Adjacent transverse sections through E5 chicken otic vesicles infected with RCAS(A)/DNErbB4-flag 48 hours earlier and processed for DAPI staining (C), anti-3C2 immunostaining (D), *DNErbB4 in situ* hybridization (E), or anti-ErbB4 immunostaining (F). The *DNErbB4 in situ* probe and the anti-ErbB4 antibody are specific to human ErbB4. 3C2-gag immunostaining reveals broad infection of the otic epithelium, including the lateral pouch (asterisk). Both *DNErbB4* transcript (E) and DNErbB4 protein (F) are produced here (asterisks). In some areas, viral infection does not generate detectable amounts of DNErbB4-flag (arrowhead). Therefore, in all subsequent experiments, the extent of infection was monitored by *in situ* hybridization for *DNErbB4*.

To block ErbB signaling in the chicken otic vesicle, we used a virus to express a truncated version of the ErbB4 receptor. This construct blocks all Neuregulin signaling by binding NRG ligands and preventing them from binding to endogenous ErbB2, ErbB3, and ErbB4 [49] (Fig. 6A). This same strategy was used to block ErbB signaling successfully in both mice and chickens [30], [50], [51]. The DNErbB4-flag construct was cloned into the RCAS vector; control viruses consisted of an RCAS virus expressing either alkaline phosphatase or a secreted form of GFP. To confirm that the RCAS(A)/DNErbB4-flag blocks ErbB signaling effectively, chicken DF1 cells were transfected with ErbB4 to make them NRG responsive. The same cells were then infected with increasing amounts RCAS(A)/DNErbB4-flag. As expected, increasing amounts of the dominant negative construct cause a dose-dependent reduction in ErbB receptor phosphorylation in response to NRG (Fig. 6B). Hence, NRG signaling is strongly blocked in the presence of RCAS(A)/DNErbB4-flag. Having assessed the efficacy of the virus in blocking ErbB signaling, we next tested the ability of RCAS(A)/DNErbB4-flag to infect chicken epithelium *in vivo.* Both *in situ* hybridization using a probe specific to the *DNErbB4* construct (Fig. 6E) and ErbB4 immunostaining of infected chicken otic vesicles sections (Fig. 6F) demonstrate that DNErbB4 mRNA and protein are produced in otic epithelium. Therefore, we conclude that RCAS(A)/DNErbB4-flag provides a suitable tool for blocking NRG signaling *in vivo*.

**Figure 7:**
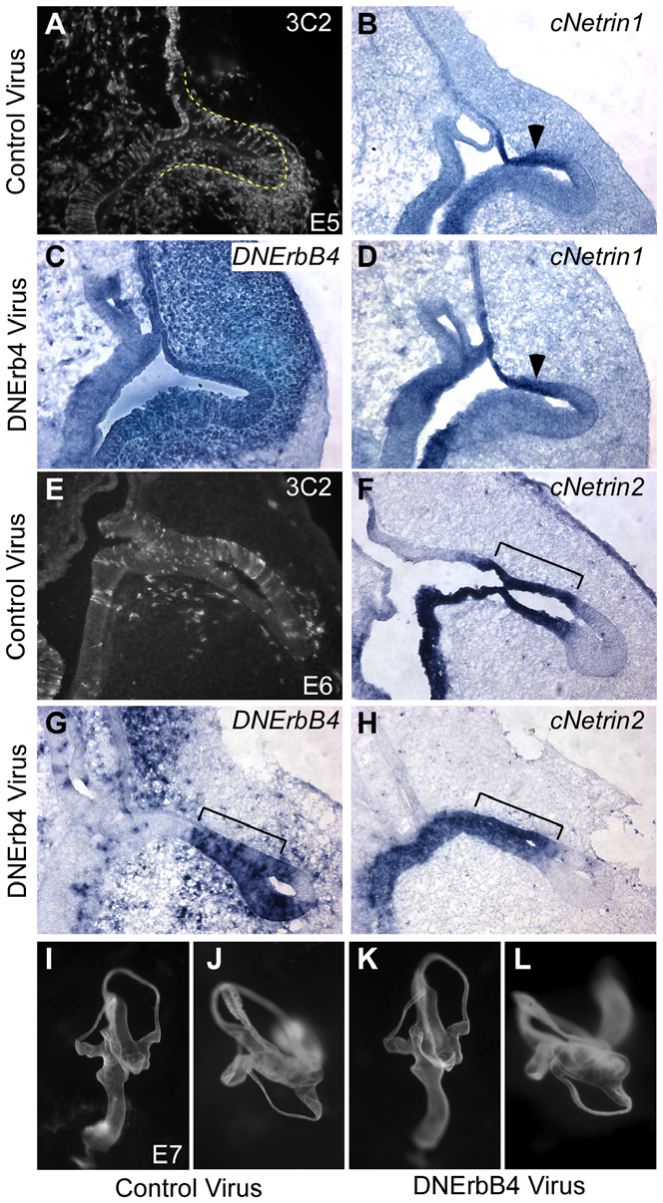
Broad expression of dominant negative ErbB has no effect on *Netrin* gene expression or canal morphogenesis. (**A–H**) Transverse sections through E5 (A–D) or E6 (E-H) chick heads infected with control (A,B,E,F) or DNErbB4 (C,D,G,H) virus. The lateral pouch is outlined in A. The extent of infection was assayed by 3C2-gag immunostaining for control virus (A,C) or by *in situ* hybridization for *DNErbB4* (C,G). Adjacent sections were probed for *cNetrin1* (B,D) or *cNetrin2* (F,H). At both E5 and E6, *cNetrin1* and *cNetrin2* are expressed normally in the fusion plate epithelium (arrowheads, brackets) despite abundant expression of *DNErbB4* here. (**I–L**) Lateral (I,K) and top-down (J,L) views of E7 paintfilled inner ears of control (I,J) and experimental (K,L) embryos. No change in the size or shape of the overall inner ear (I,L) or lateral canal (J,L) is evident.

We next assayed whether inhibition of NRG signaling has any effect on *Netrin* transcription or canal morphogenesis. RCAS(A)/DNErbB4-flag (5.6×10^9^ iu/ml) or control virus (RCAS(A)/GFP at 2×10^9^ iu/ml or RCAS(A)/AP at 5×10^8^ iu/ml) were injected into the otic vesicles of E3 chickens, allowed to infect for 72 hours and then sacrificed to assess the level of infection by immunostaining against the 3C2-gag viral coat protein (Fig. 7A,C,E,G). Since patterns of RCAS infection vary from embryo to embryo and not all infected cells produce the protein of interest, we also confirmed expression of *DNErbB4*. Consistent with the broad expression of 3C2-gag in the mesenchyme and epithelium, *DNErbB4-flag* is widely misexpressed in the lateral pouch epithelium at E5 (Fig. 7C). However, no change in *cNetrin1* is detected either at E5 (Fig.7B,D) or E6 (data not shown). Similarly, high levels of *cNetrin2* are maintained throughout the fusion plate at E6 despite abundant expression of DNErbB4-flag throughout the lateral pouch epithelium (Fig.7F,H) (n = 10 DNErbB4 infected and 7 control infected embryos for *cNetrin1* and n = 9 DNErbB4 infected and 8 control infected embryos for *cNetrin2*). To assess whether reduced ErbB signaling in the developing otic vesicle may result in canal defects independent of changes in *Netrin* expression, some injected chickens were allowed to develop until E7, when canal morphogenesis is complete. However, paintfills reveal no differences in the shape or size of the canals in RCAS(A)/DNErbB4-flag infected inner ears (n = 10) when compared to controls (n = 8) (Fig. 7I–L). There are other sites of ErbB/Lrig3 overlap outside of the lateral pouch, so it is possible that blocking ErbB signaling could result in other defects of inner ear development that paintfills cannot reveal. In addition, since our approach does not remove all ErbB activity, we cannot rule out that transient and/or low levels of NRG signaling persist and are sufficient for perfect formation of the inner ear. However, since *DNErbB4* was broadly expressed (i.e. Fig. 7C,G) and was highly effective *in vitro* (Fig. 6B), these experiments suggest that misregulation of NRG signaling is unlikely to explain the *Lrig3* canal defects.

## Discussion

Although Lrig proteins harbor common protein-protein interaction motifs in their extracellular domain, their cytoplasmic domains are widely divergent and specific molecular functions have remained elusive. Here, we confirm a general ability for Lrig proteins to interact with ErbB receptors *in vitro*. However, misexpression of dominant negative ErbB4 has no effect on *Netrin* expression or the structure of the inner ear, suggesting that aberrant NRG signaling is not a good explanation for Lrig3-dependent aspects of canal morphogenesis. These results add to the mounting evidence that Lrig proteins are not dedicated regulators of ErbB signaling; rather, they may have multiple functions. Our findings highlight the incomplete knowledge of Lrig protein function at present.

Since Lrig proteins have highly conserved extracellular domains with many protein-protein interaction motifs, a better understanding of the nature and variety of *bona fide* binding partners will provide important insights into Lrig function. Although ErbB proteins were the first binding partners to be identified, Lrig proteins are also able to interact with other receptor tyrosine kinases that share no homology with the ErbB ectodomain. For instance, Lrig1 can also inhibit Met and Ret signaling pathways, while Lrig3 has been shown to bind to the FGF receptor [52]–[54]. In addition, our own *in vitro* studies have shown that in addition to the ErbB receptors, Lrig3 is also able to bind to a wide variety of receptors, including the Netrin1 receptors Unc5Ha-c, the Neurotrophin receptor p75 and the axon guidance receptor PlexinA1 (data not shown). Our discovery that the *Lrig3* mutant phenotype is apparently unrelated to NRG signaling emphasizes the need to confirm the relevance of each of these *in vitro* binding interactions for specific *in vivo* functions.

Although Lrig1 and Lrig3 may share the ability to interact with a wide variety of binding partners *in vitro*, whether or not this reflects similar cell biological functions *in vivo* remains unclear. Although the extracellular domains of Lrig3 and Lrig1 are highly conserved, the proteins share very little homology in their intracellular domains. For example, Lrig3 shows no similarity to Lrig1 in the portion of the cytoplasmic domain that binds to c-Cbl. Consistent with this observation, although Lrig3 is able to bind to ErbB receptors, we noticed that unlike Lrig1, Lrig3 does not induce a dramatic downregulation of ErbB receptor levels (see Fig. 2A). Rather, there is a trend towards increased ErbB receptor levels, suggesting that Lrig3/ErbB interactions may not result in efficient degradation at all. Thus, Lrig3's ability to bind ErbB receptors does not necessarily imply that Lrig3 also induces ubiquitination of ErbB receptors. Indeed, even Lrig1 exhibits c-Cbl independent functions, since Lrig1-mediated degradation of the Met receptor does not involve polyubiquitination [53]. Moreover, Lrig1 inhibits Ret function by preventing recruitment to lipid rafts, with no effect on internalization [52]. Such examples highlight the possibility that individual Lrig proteins may have diverse molecular and cellular functions depending on the context.

To date, investigations of *Lrig1* and *Lrig3* mutant mouse phenotypes have confirmed that both molecules are important for aspects of development, but have done little to clarify their specific functions [24], [27]. Although Lrig1 has been proposed to act through ErbB in the skin, whereas Lrig3 does not seem to function through NRG signaling in the inner ear, our results do not rule out shared functions for Lrig1 and Lrig3 in other tissues. Indeed, based on the broad and overlapping expression patterns for *Lrig1* and *3* during development, the limited number of phenotypes evident in each mouse mutant suggests that Lrig1 and 3 may function together. Our studies provide additional evidence that Lrig1 and Lrig3 can in fact act through common pathways that have yet to be defined. For instance, Lrig1 and Lrig3 have similar binding properties, raising the possibility that Lrig3 could influence Lrig1's ability to induce receptor internalization. These similarities with respect to ErbB signaling *in vitro* could also reflect a basic binding property that enables interactions with another signaling pathway that has yet to be defined. The nature of any additional defects that arise in *Lrig1/Lrig3* double mutant mice is likely to provide useful insights into this question in the future.

Since ErbB receptors do not seem to be involved in the *Lrig3*-*Netrin* feedback loop, the identity of the RTK hypothesized to be inhibited by Lrig3 remains unclear. The best candidate is the FGF receptor, not only because Lrig3 is known to bind to and inhibit FGF receptor [54], but also because of the known importance of FGF signaling during canal morphogenesis [45], [55]–[57]. FGF signaling promotes the formation of the fusion plate and the proliferation of the periotic mesenchyme [57]. Although FGF ligands and receptors have been identified in several different regions of the developing inner ear, uncovering a possible Lrig/FGFR interaction in the inner ear will be difficult, as multiple feedback-induced antagonists are present and may mask Lrig activities.

As well as revealing the need to look beyond ErbB receptors as biologically relevant binding partners for Lrig3, our studies provide evidence that NRG signaling is not essential for canal morphogenesis. This result is unexpected given the early and broad expression of ErbB2/B3 in the otic epithelium as well as the prominent role for ErbB activation elsewhere in the embryo. As with any negative result, it is possible that the absence of a phenotype is due to the technical limitations of the experiment, since low level NRG signaling could persist in these conditions. However, we consider this unlikely for the following reasons. First, this virus effectively blocks NRG signaling in chicken cells (Fig. 6). Second, we achieved broad expression of DNErbB4 throughout the otic epithelium (Fig. 7). Third, since DNErbB4 works by binding to and sequestering the NRG ligand, NRG signaling will be reduced not only in DNErbB4 expressing cells, but also in the surrounding tissue. Fourth, the inner ear is extremely sensitive to even modest changes in signaling, as evidenced by the presence of defects in several heterozygous mouse lines [46], [58]. Nonetheless, we did not detect even a subtle change in canal structure or *Netrin* expression. Finally, although *ErbB2* has been shown to be essential for normal innervation in the inner ear, no canal defects were reported [59]. Thus, we favor the interpretation that NRG signaling is not essential for canal morphogenesis.

Nevertheless, it is possible that NRG signaling is important for other aspects of ear development that may also involve Lrig1 and/or Lrig3. Our assays were designed to detect changes in canal structure and *cNetrin1/2* gene expression, and would not have revealed cellular phenotypes in the sensory epithelia or other regions of the inner ear. For example, *Lrig1* and *Lrig3* appear to overlap with *ErbB2-4* in the neonatal cochlea [29], [60], where they might fine tune NRG-induced production of BDNF and subsequent neuronal survival. Indeed, inhibition of NRG signaling in cochlear support cells by the same construct used here causes spiral ganglion neuron degeneration and hearing deficits [30]. Thus, while our results rule out a role in canal morphogenesis, Lrig-ErbB interactions may influence other aspects of inner ear development and function. If these activities are uncovered in future investigations of *Lrig3* or *Lrig1;Lrig3* mutant animals, more detailed investigations of Lrig3-ErbB interactions would be warranted.

A deeper understanding of Lrig protein activities will be critical for elucidating the relationship between Lrig proteins and cancer. All three family members have been implicated in a broad range of cancers, either because the gene is deleted or the protein changes its localization [14]–[23]. Given the prominent role for NRG signaling in cancer, it has been often suggested that the loss of Lrig1 enhances tumor progression by inducing overactivation of the ErbB pathway [4], [17], [18]. However, our results suggest that this is an oversimplified view of Lrig function. It is possible that many other receptor tyrosine kinase pathways are also misregulated in the absence of Lrig1, raising the possibility that ErbB-targeted drugs might not be appropriate in these cases. Conversely, activation of Lrig with new drugs may not be a viable option for slowing tumor development. Through continued dissection of the signaling properties of this intriguing protein family both *in vitro* and *in vivo*, the full extent of Lrig's contribution to development and disease will become clear.

## Materials and Methods

### Plasmids and HEK Transfections

The human N'flag-Lrig1 construct was a gift from H. Hedman (Umea University, Umea, Sweden). The ErbB4 and DNErbB4-flag constructs were kindly provided by G. Corfas (Children's Hospital, Boston). Full length cDNA from mouse *Lrig3* was cloned into pcDNA3.1+, and a flag tag was cloned into the unique SmaI site immediately following the signal sequence; the epitope with flanking protein sequence is HGAPGMDYKDDDDKGQLLDD. HEK293T cells were cultured in Dulbecco's Minimal Essential Medium (DMEM) (Invitrogen) containing 10% fetal bovine serum and 1% penicillin/streptomycin. Cells were plated at 60–70% confluency on six-well or 10 cm plates, transfected using FuGene6 (Roche), and harvested 24 hours later. When indicated, cells were starved in DMEM without serum for 16 hours and treated with 1 nM recombinant human Neuregulin1-b1 (NRG1, R&D Systems) or 200 ng/ml of Human EGF (Protech) for 10 min before lysis.

### Co-Immunoprecipitation

Cells were washed with cold phosphate buffered saline (PBS) and lysed in 20 mM Tris (pH 7.4), 150 mM NaCl, 1 mM EDTA, 1 mM EGTA, 1% TritonX-100, 2.5 mM sodium pyrophosphate, 1 mM βglycerol phosphate, 1 mM Na_3_VO_4_, and 1 mM of Pefabloc (Roche). For immunoprecipitations, lysates were pre-cleared with agarose-conjugated normal IgG for 30 min at 4°C and immunoprecipitated with agarose conjugated flag antibody (M2, Sigma) overnight at 4°C. Samples were washed four times with lysis buffer before the beads were resuspended in SDS sample buffer and boiled for 5 min. Western analysis was performed using standard protocols and the following antibodies: EGFR (1∶1000, Santa Cruz), ErbB2 (1∶1000, Abcam), ErbB3 (1∶1000, Santa Cruz), ErbB4 (1∶1000, Santa Cruz); flag (1∶1000 M2, Sigma), and actin (1∶8000, Abcam).

### Immunocytochemistry

Cells grown on glass coverslips were fixed with 4% paraformaldehyde (PFA) in PBS for 10 min on ice, permeabilized in 0.1%Triton X-100 for 10 min, blocked with 5% bovine serum albumin (BSA) for 30 min, and then incubated overnight at 4°C with primary antibodies diluted in blocking solution: flag (1∶1000, M2 Sigma) and ErbB4 (1∶500, Santa Cruz). The coverslips were washed with PBS, and detection was performed using appropriate secondary antibodies (1∶2000, Jackson Immunoresearch) for 1 hour at room temperature. Nuclei were counterstained with DAPI for 5 min (1∶10,000, Sigma), washed with PBS, and mounted with Vectashield (Vector Laboratories).

### Mice

*Lrig3^flox^* conditional mice [27] and *Pax2Cre* mice [32] have been maintained for over five generations on C57Bl6/J background. *Rosa26 reporter* mice (*R26R*) [61] and *Z/EG* reporter [62] have been maintained for over five generations on a CD1 background. Genotyping was performed as described or using primers to amplify Cre [297 (ATTTGCCTGCATTACCGGTC) and 298 (ATCAACGTTTTCTTTTCGGA)] or GFP [7110 (TACGGCAAGCTGACCCTGAAGTTC) and 7111 (AAGTCGATGCCCTTCAGCTCGATG)]. All mice are maintained in accordance with institutional and NIH guidelines approved by the IACUC at Harvard Medical School.

### *In Situ* Hybridization

Non-radioactive *in situ* hybridization was performed on 12–14 µm frozen sections using the following probes: Mouse: *EGFR* (ENSMUST00000020329, nt3816–4429) and *ErbB3* (ENSMUST00000020329, nt4230–4800); Chicken: *cLrig3* (ENSGALG00000009755, nt2931–3643), *cErbB2* (NM_001044661.1, nt2235–2934), *cErbB3* (NM_001044669.1, nt2173–2880), *cNetrin2* (L34550, nt2097–2645). The chicken *Netrin1* probe was provided by C. Cepko (Harvard Medical School), and the *DNErbB4* probe was a gift from G. Corfas (Children's Hospital, Boston). A detailed protocol is available at http://goodrich.med.harvard.edu/.

### Otic Vesicle Western Blots

*Pax2Cre;Z/EG* otic vesicles were microdissected from E12 embryos and lysed as described above. The samples were homogenized on ice for 10 min, and then centrifuged at 1000×g for 10 min at 4°C. Supernatant was either used immediately for Western analysis or frozen in SDS sample buffer at −80°C. Western analysis was performed as described above using the following antibodies: EGFR (1∶1000, Santa Cruz), ErbB2 (1∶1000, Abcam), ErbB3 (1∶1000, Santa Cruz), ErbB4 (1∶1000, Santa Cruz).

### X-Gal Staining

Staining for β−galactosidase was performed as described [63] except that 10–20 µm frozen sections were used, and the tissue was fixed for 1 hour at 4°C.

### Construction, Production and Validation of RCAS(A)/DNErbB4 Virus

A Replication-Competent Avian sarcoma-leukosis retroviral vector, RCAS(A), containing the DNErbB4-flag construct was built using Gateway recombination cloning into the RCASBP-Y DV destination vector [64]. Specifically, attB1 and attB2 sites were added to the ends of DNerbB4-flag fragment using PCR with primers B1F_DNEB (5′ GGGGACAAGTTTGTACAAAAAAGCAGGCTGAACCATGATGAAGCCGGCGACAGGACT 3′) and B2R_DNEB (5′ GGGGACCACTTTGTACAAGAAAGCTGGGTCTCACTTGTCG-TCATCGTCTTTG 3′). The PCR fragments were first inserted into pDONR221, using BP Clonase II (Invitrogen), resulting in an entry vector, pME-DNEB. The DNerbB4-flag fragment in pME-DNEB was cloned into RCASBP-Y DV using LR Clonase II (Invitrogen) to create pRCAS-DNErbB4 proviral vector. RCAS/DNErbB4-flag virus stock was generated in UMNSAH/DF-1 chicken fibroblasts (ATCC CRL-12203), concentrated by centrifugation and titered on these cells as described previously [65].

RCAS(A)/DNErbB4-flag was used at a titer of 5.6×10^9^ infectious units/ml (iu/ml). Control viruses included an RCAS(A)/GFP at a titer of 2×10^9^ infectious units/ml and RCAS(A)/AP at a titer of 5×10^8^ infectious units/ml. To validate that RCAS(A)/DNErbB4-flag blocks NRG signaling, DF1 chicken cells were grown to ∼30% confluency and transiently transfected with ErbB4 using FuGene6 (Invitrogen). The next day, each well was infected with either no virus, 5.6×10^5^ iu/ml or 5.6×10^6^ iu/ml of virus for an hour while rocking at 37°C. 24 hours post-infection, cells were serum starved for an additional 16 hours and then treated with 1 nM recombinant human Neuregulin1-b1 (NRG1, R&D Systems) for 10 minutes before lysis. Western analysis of the cell lysates was performed with the following antibodies: phosphotyrosine (1∶1000, Upstate), flag (1∶1000, M2, Sigma); ErbB4 (1∶1000, C-18, Santa Cruz); and actin (1∶8000, Abcam). Chicken DF1 cells were maintained in DMEM (Invitrogen) with 10% FBS, 5% chicken serum, and 1% penicillin/streptomycin.

### Viral Infection of Chick Otic Vesicles

White leghorn premium quality chicken eggs were maintained in a 39°C humidified incubator for the duration of the experiment (7 days), according to IACUC guidelines at Harvard Medical School. RCAS(A)/DNErbB4-flag virus or control virus was injected into the right otic vesicle of E3 (stage 17) chick embryos. In order to visualize the amount of virus injected, 1–5 µl of 0.25% Fast Green solution was combined with 10–50 µl of virus. Using a backloading pipette tip (Eppendorf), 5 µl of the virus with the Fast Green solution was loaded into a glass pipette that had been previously prepared by pulling Omega dot capillaries (No. 30-30-0 1.0 mm od; 0.75 mm id; 100 mm long; FHC, Brunswick, ME). To inject the virus, we used a picospritzer needle holder attached to a micromanipulator (Picospritzer: Picospritzer III from Parker Ins; micromanipulator: Siskiyou Instruments; stand: Fisso S-20 from Flexbar). Tungsten needle and forceps were used to open the vitelline membrane that surrounds the head of the embryo. The loaded pipette tip was inserted into the otic vesicle using the micromanipulator, and with the picrospritzer, the virus was injected to fill the entire otic vesicle. Following a successful injection the egg was sealed and placed back into the humidified incubator until E5, E6, or E7.

### Immunohistochemistry

E5 chick embryos were collected and fixed for 1–2 hours at 4°C in 4% PFA/PBS and then dehydrated in 30% sucrose/PBS overnight at 4°C and equilibrated in Neg50 (Richard-Allan Scientific) for 2–3 hours at 4°C, followed by embedding in Neg50. Frozen sections (10–14 µms) were blocked and permeabilized in 5% normal donkey serum +2% BSA +0.1% Triton X-100 in PBS for 1 hour at room temperature. Primary antibodies were added into the above block, without Triton X-100, overnight at 4°C at the following concentrations: 3C2-gag protein (1∶10, DSHB) and c-ErbB4 (1∶250, H4.77.16 Neomarkers). The following day, the sections were incubated in Alexa Fluor488 or Alexa Fluor568 antibody (1∶2000, Jackson Immunoresearch) in block, without Triton X-100. All sections were counterstained with DAPI (1∶10,000).

### Paintfills

E7 chick or E14 mouse heads were fixed overnight at 4°C with Bodian's Fix, dehydrated overnight at room temperature with 100% ethanol, then cleared overnight at room temperature with methyl salicylate. Heads were hemisected, and white latex paint (Benjamin Moore) diluted to 0.025% in methyl salicylate was injected into the cochlea with a pulled glass pipette and a Hamilton syringe filled with glycerol.

## References

[1] Dolan J, Walshe K, Alsbury S, Hokamp K, O'Keeffe S (2007). The extracellular leucine-rich repeat superfamily; a comparative survey and analysis of evolutionary relationships and expression patterns.. BMC Genomics.

[2] Ghiglione C, Amundadottir L, Andresdottir M, Bilder D, Diamonti JA (2003). Mechanism of inhibition of the Drosophila and mammalian EGF receptors by the transmembrane protein Kekkon 1.. Development.

[3] Ghiglione C, Carraway KL, Amundadottir LT, Boswell RE, Perrimon N (1999). The transmembrane molecule kekkon 1 acts in a feedback loop to negatively regulate the activity of the Drosophila EGF receptor during oogenesis.. Cell.

[4] Goldoni S, Iozzo RA, Kay P, Campbell S, McQuillan A (2007). A soluble ectodomain of LRIG1 inhibits cancer cell growth by attenuating basal and ligand-dependent EGFR activity.. Oncogene.

[5] Gur G, Rubin C, Katz M, Amit I, Citri A (2004). LRIG1 restricts growth factor signaling by enhancing receptor ubiquitylation and degradation.. Embo J.

[6] Laederich MB, Funes-Duran M, Yen L, Ingalla E, Wu X (2004). The leucine-rich repeat protein LRIG1 is a negative regulator of ErbB family receptor tyrosine kinases.. J Biol Chem.

[7] Burden S, Yarden Y (1997). Neuregulins and their receptors: a versatile signaling module in organogenesis and oncogenesis.. Neuron.

[8] Miyazaki Y, Nakanishi Y, Hieda Y (2004). Tissue interaction mediated by neuregulin-1 and ErbB receptors regulates epithelial morphogenesis of mouse embryonic submandibular gland.. Dev Dyn.

[9] Dammann CE, Nielsen HC, Carraway KL (2003). Role of neuregulin-1 beta in the developing lung.. Am J Respir Crit Care Med.

[10] Camenisch TD, Schroeder JA, Bradley J, Klewer SE, McDonald JA (2002). Heart-valve mesenchyme formation is dependent on hyaluronan-augmented activation of ErbB2-ErbB3 receptors.. Nat Med.

[11] Woldeyesus MT, Britsch S, Riethmacher D, Xu L, Sonnenberg-Riethmacher E (1999). Peripheral nervous system defects in erbB2 mutants following genetic rescue of heart development.. Genes Dev.

[12] Carraway KL, Carraway CA, Carraway KL (1997). Roles of ErbB-3 and ErbB-4 in the physiology and pathology of the mammary gland.. J Mammary Gland Biol Neoplasia.

[13] LeBras S, Czernichow P, Scharfmann R (1998). A search for tyrosine kinase receptors expressed in the rat embryonic pancreas.. Diabetologia.

[14] Hedman H, Nilsson J, Guo D, Henriksson R (2002). Is LRIG1 a tumour suppressor gene at chromosome 3p14.3?. Acta Oncol.

[15] Lindstrom AK, Ekman K, Stendahl U, Tot T, Henriksson R (2007). LRIG1 and squamous epithelial uterine cervical cancer: correlation to prognosis, other tumor markers, sex steroid hormones, and smoking.. Int J Gynecol Cancer.

[16] Ljuslinder I, Golovleva I, Palmqvist R, Oberg A, Stenling R (2007). LRIG1 expression in colorectal cancer.. Acta Oncol.

[17] Yang WM, Yan ZJ, Ye ZQ, Guo DS (2006). LRIG1, a candidate tumour-suppressor gene in human bladder cancer cell line BIU87.. BJU Int.

[18] Miller JK, Shattuck DL, Ingalla EQ, Yen L, Borowsky AD (2008). Suppression of the negative regulator LRIG1 contributes to ErbB2 overexpression in breast cancer.. Cancer Res.

[19] Tanemura A, Nagasawa T, Inui S, Itami S (2005). LRIG-1 provides a novel prognostic predictor in squamous cell carcinoma of the skin: immunohistochemical analysis for 38 cases.. Dermatol Surg.

[20] Xiong Z, Cao Y, Guo D, Ye F, Lei T (2006). Expression of EGFR and LRIG-1 in human trigeminal neurinoma.. J Huazhong Univ Sci Technolog Med Sci.

[21] Guo D, Nilsson J, Haapasalo H, Raheem O, Bergenheim T (2006). Perinuclear leucine-rich repeats and immunoglobulin-like domain proteins (LRIG1-3) as prognostic indicators in astrocytic tumors.. Acta Neuropathol (Berl).

[22] Hedman H, Henriksson R (2007). LRIG inhibitors of growth factor signalling - double-edged swords in human cancer?. Eur J Cancer.

[23] Holmlund C, Nilsson J, Guo D, Starefeldt A, Golovleva I (2004). Characterization and tissue-specific expression of human LRIG2.. Gene.

[24] Suzuki Y, Miura H, Tanemura A, Kobayashi K, Kondoh G (2002). Targeted disruption of LIG-1 gene results in psoriasiform epidermal hyperplasia.. FEBS Lett.

[25] Jensen KB, Watt FM (2006). Single-cell expression profiling of human epidermal stem and transit-amplifying cells: Lrig1 is a regulator of stem cell quiescence.. Proc Natl Acad Sci U S A.

[26] Pastore S, Mascia F, Mariani V, Girolomoni G (2008). The epidermal growth factor receptor system in skin repair and inflammation.. J Invest Dermatol.

[27] Abraira VE, Del Rio T, Tucker AF, Slonimsky J, Keirnes HL (2008). Cross-repressive interactions between Lrig3 and netrin 1 shape the architecture of the inner ear.. Development.

[28] Salminen M, Meyer BI, Bober E, Gruss P (2000). Netrin 1 is required for semicircular canal formation in the mouse inner ear.. Development.

[29] Hume CR, Kirkegaard M, Oesterle EC (2003). ErbB expression: the mouse inner ear and maturation of the mitogenic response to heregulin.. J Assoc Res Otolaryngol.

[30] Stankovic K, Rio C, Xia A, Sugawara M, Adams JC (2004). Survival of adult spiral ganglion neurons requires erbB receptor signaling in the inner ear.. J Neurosci.

[31] Carraway KL, Ramsauer VP, Carraway CA (2005). Glycoprotein contributions to mammary gland and mammary tumor structure and function: roles of adherens junctions, ErbBs and membrane MUCs.. J Cell Biochem.

[32] Ohyama T, Groves AK (2004). Generation of Pax2-Cre mice by modification of a Pax2 bacterial artificial chromosome.. Genesis.

[33] Erickson SL, O'Shea KS, Ghaboosi N, Loverro L, Frantz G (1997). ErbB3 is required for normal cerebellar and cardiac development: a comparison with ErbB2-and heregulin-deficient mice.. Development.

[34] Lin W, Sanchez HB, Deerinck T, Morris JK, Ellisman M (2000). Aberrant development of motor axons and neuromuscular synapses in erbB2-deficient mice.. Proc Natl Acad Sci U S A.

[35] Morris JK, Lin W, Hauser C, Marchuk Y, Getman D (1999). Rescue of the cardiac defect in ErbB2 mutant mice reveals essential roles of ErbB2 in peripheral nervous system development.. Neuron.

[36] Qu S, Rinehart C, Wu HH, Wang SE, Carter B (2006). Gene targeting of ErbB3 using a Cre-mediated unidirectional DNA inversion strategy.. Genesis.

[37] Wong RW (2003). Transgenic and knock-out mice for deciphering the roles of EGFR ligands.. Cell Mol Life Sci.

[38] Gilmore JL, Scott JA, Bouizar Z, Robling A, Pitfield SE (2008). Amphiregulin-EGFR signaling regulates PTHrP gene expression in breast cancer cells.. Breast Cancer Res Treat.

[39] Fowler KJ, Walker F, Alexander W, Hibbs ML, Nice EC (1995). A mutation in the epidermal growth factor receptor in waved-2 mice has a profound effect on receptor biochemistry that results in impaired lactation.. Proc Natl Acad Sci U S A.

[40] Lee KF, Simon H, Chen H, Bates B, Hung MC (1995). Requirement for neuregulin receptor erbB2 in neural and cardiac development.. Nature.

[41] Riethmacher D, Sonnenberg-Riethmacher E, Brinkmann V, Yamaai T, Lewin GR (1997). Severe neuropathies in mice with targeted mutations in the ErbB3 receptor.. Nature.

[42] Gassmann M, Casagranda F, Orioli D, Simon H, Lai C (1995). Aberrant neural and cardiac development in mice lacking the ErbB4 neuregulin receptor.. Nature.

[43] Martin P, Swanson GJ (1993). Descriptive and experimental analysis of the epithelial remodellings that control semicircular canal formation in the developing mouse inner ear.. Dev Biol.

[44] Bissonnette JP, Fekete DM (1996). Standard atlas of the gross anatomy of the developing inner ear of the chicken.. J Comp Neurol.

[45] Chang W, Brigande JV, Fekete DM, Wu DK (2004). The development of semicircular canals in the inner ear: role of FGFs in sensory cristae.. Development.

[46] Chang W, Lin Z, Kulessa H, Hebert J, Hogan BL (2008). Bmp4 is essential for the formation of the vestibular apparatus that detects angular head movements.. PLoS Genet.

[47] Kennedy TE, Serafini T, de la Torre JR, Tessier-Lavigne M (1994). Netrins are diffusible chemotropic factors for commissural axons in the embryonic spinal cord.. Cell.

[48] Serafini T, Kennedy TE, Galko MJ, Mirzayan C, Jessell TM (1994). The netrins define a family of axon outgrowth-promoting proteins homologous to C. elegans UNC-6.. Cell.

[49] Rio C, Rieff HI, Qi P, Khurana TS, Corfas G (1997). Neuregulin and erbB receptors play a critical role in neuronal migration.. Neuron.

[50] Golding JP, Sobieszczuk D, Dixon M, Coles E, Christiansen J (2004). Roles of erbB4, rhombomere-specific, and rhombomere-independent cues in maintaining neural crest-free zones in the embryonic head.. Dev Biol.

[51] Sakakibara A, Horwitz AF (2006). Mechanism of polarized protrusion formation on neuronal precursors migrating in the developing chicken cerebellum.. J Cell Sci.

[52] Ledda F, Bieraugel O, Fard SS, Vilar M, Paratcha G (2008). Lrig1 is an endogenous inhibitor of Ret receptor tyrosine kinase activation, downstream signaling, and biological responses to GDNF.. J Neurosci.

[53] Shattuck DL, Miller JK, Laederich M, Funes M, Petersen H (2007). LRIG1 is a novel negative regulator of the Met receptor and opposes Met and Her2 synergy.. Mol Cell Biol.

[54] Zhao H, Tanegashima K, Ro H, Dawid IB (2008). Lrig3 regulates neural crest formation in Xenopus by modulating Fgf and Wnt signaling pathways.. Development.

[55] Pauley S, Wright TJ, Pirvola U, Ornitz D, Beisel K (2003). Expression and function of FGF10 in mammalian inner ear development.. Dev Dyn.

[56] Pirvola U, Spencer-Dene B, Xing-Qun L, Kettunen P, Thesleff I (2000). FGF/FGFR-2(IIIb) signaling is essential for inner ear morphogenesis.. J Neurosci.

[57] Pirvola U, Zhang X, Mantela J, Ornitz DM, Ylikoski J (2004). Fgf9 signaling regulates inner ear morphogenesis through epithelial-mesenchymal interactions.. Dev Biol.

[58] Alavizadeh A, Kiernan AE, Nolan P, Lo C, Steel KP (2001). The Wheels mutation in the mouse causes vascular, hindbrain, and inner ear defects.. Dev Biol.

[59] Morris JK, Maklad A, Hansen LA, Feng F, Sorensen C (2006). A disorganized innervation of the inner ear persists in the absence of ErbB2.. Brain Res.

[60] Zhang M, Ding D, Salvi R (2002). Expression of heregulin and ErbB/Her receptors in adult chinchilla cochlear and vestibular sensory epithelium.. Hear Res.

[61] Soriano P (1999). Generalized lacZ expression with the ROSA26 Cre reporter strain.. Nat Genet.

[62] Novak A, Guo C, Yang W, Nagy A, Lobe CG (2000). Z/EG, a double reporter mouse line that expresses enhanced green fluorescent protein upon Cre-mediated excision.. Genesis.

[63] Leighton PA, Mitchell KJ, Goodrich LV, Lu X, Pinson K (2001). Defining brain wiring patterns and mechanisms through gene trapping in mice.. Nature.

[64] Loftus SK, Larson DM, Watkins-Chow D, Church DM, Pavan WJ (2001). Generation of RCAS vectors useful for functional genomic analyses.. DNA Res.

[65] Morgan BA, Fekete DM (1996). Manipulating gene expression with replication-competent retroviruses.. Methods Cell Biol.

